# Association between the triglyceride glucose-waist circumference index and cardiovascular disease across different glycemic statuses among middle-aged and older Chinese adults

**DOI:** 10.3389/fcvm.2025.1608655

**Published:** 2025-07-14

**Authors:** Yuyu Cui, Zhening Xu, Lijuan Ding, Yanju Li, Xiaoyan Zhou, Lingxia Li

**Affiliations:** ^1^ Department of Physiology and Pathophysiology, Yan'an Medical College of Yan'an University, Yan'an, Shaanxi Province, China; ^2^ Department of Anesthesiology, Affiliated Hospital of Yan'an University, Yan'an, Shaanxi Province, China

**Keywords:** triglyceride glucose-waist circumference, cardiovascular disease, prediabetes, diabetes mellitus, China health and retirement longitudinal study

## Abstract

**Introduction:**

This study aims to systematically investigate the association between the triglyceride-glucose index multiplied by waist circumference (TyG-WC) and the risk of cardiovascular disease (CVD) and further explore how this relationship varies across different glycemic statuses, including normal glucose regulation (NGR), prediabetes (Pre-DM), and diabetes mellitus (DM).

**Methods:**

Data were obtained from the China Health and Retirement Longitudinal Study (CHARLS), including a total of 7,812 middle-aged and older adults. Kaplan–Meier survival analysis, multivariable Cox proportional hazards models, and restricted cubic spline (RCS) regression were employed to assess the association between baseline TyG-WC and incident CVD risk. Subgroup analyses were conducted based on glucose metabolism status to evaluate potential heterogeneity in the associations.

**Result:**

During an average follow-up period of 8.25 years, a total of 1,638 incident CVD events were recorded, corresponding to a cumulative incidence of 20.97%. Kaplan–Meier curves showed that individuals in higher TyG-WC strata had significantly greater cumulative CVD incidence compared to those in lower strata across all glucose metabolism categories (log-rank test, *P* < 0.05). After adjusting for potential confounders, the hazard ratios (HRs) [95% confidence intervals (CIs)] for CVD in the second, third, and fourth TyG-WC quartiles (Q2–Q4) were 1.20 (1.05–1.37), 1.30 (1.14–1.49), and 1.54 (1.34–1.77), respectively, compared to Q1. In the NGR and Pre-DM groups, TyG-WC was positively and linearly associated with CVD risk. In contrast, a significant non-linear association was observed in the DM group (*P* for non-linear = 0.046). Specifically, TyG-WC was positively associated with CVD risk when values were below 816.16, whereas above this threshold the increased risk plateaued and was no longer statistically significant.

**Conclusion:**

TyG-WC is a practical and effective metabolic indicator for evaluating CVD risk among middle-aged and older Chinese adults. Its clinical application may facilitate early identification and precise stratification of high-risk individuals, thereby providing strong support for CVD prevention and targeted intervention strategies.

## 1 Introduction

Cardiovascular disease (CVD) remains one of the leading causes of mortality worldwide, with incidence and mortality rates continuing to rise, placing a substantial burden on global public health systems (1). In 2022, the global prevalence of CVD reached approximately 523 million, representing a 63% increase in CVD-related deaths compared to 1990, totaling 19.8 million fatalities (2). Therefore, the development of low-cost, reproducible, and easily deployable biomarkers to facilitate the early identification of individuals at high risk for CVD has become an urgent priority in both clinical and public health settings.

Insulin resistance (IR) is a central pathophysiological mechanism underlying type 2 diabetes, obesity, and increased susceptibility to CVD (3–7). Although the hyperinsulinemic-euglycemic clamp (HEC) technique is widely recognized as the gold standard for assessing IR, its application is limited by its complexity, time requirements, and high cost, making it impractical for large-scale population studies (8). The homeostasis model assessment of insulin resistance (HOMA-IR) is more commonly used due to its simplicity, but it relies on fasting insulin measurements, which may be inaccurate in individuals receiving insulin therapy or those with impaired β-cell function, and remains largely inaccessible in primary care settings in many regions (9). In recent years, the triglyceride–glucose (TyG) index—calculated from routine fasting glucose and triglyceride levels—has attracted considerable attention due to its simplicity, affordability, and accessibility (10, 11). Several studies have shown that TyG and its derivatives correlate more strongly with the M value from HEC than HOMA-IR and demonstrate superior predictive ability for IR in specific populations. For example, Guerrero-Romero et al. reported a correlation coefficient of −0.68 between TyG and M value, compared to −0.52 for HOMA-IR. Similarly, Zhang et al. found that in a Chinese cohort, the area under the curve (AUC) of TyG-WC for predicting IR was 0.62, higher than 0.56 for HOMA-IR (11, 12). Beyond its relationship with IR, cohort studies have demonstrated that TyG and its derivatives are strongly associated with the incidence, severity, and long-term outcomes of CVD (13–16). Composite indices such as TyG-WC and TyG-BMI may provide more sensitive reflections of IR than TyG alone (17, 18), with TyG-WC in particular showing superior predictive performance for CVD risk in Asian populations (19, 20). Although emerging evidence suggests a potential link between TyG-WC and CVD risk, most existing studies have been conducted in Western populations, and research on Asian—especially Chinese—adults remains limited. Moreover, no prior studies have systematically examined whether the association between TyG-WC and CVD varies across different glycemic statuses, such as normal glucose regulation (NGR), prediabetes (Pre-DM), and diabetes mellitus (DM). Given that glycemic status itself is a major risk factor for CVD, it may serve as an effect modifier in the relationship between TyG-WC and CVD. Clarifying this potential interaction could enhance risk stratification strategies and improve the efficiency and precision of preventive interventions. To address these gaps, we conducted a prospective analysis using nationally representative data from the China Health and Retirement Longitudinal Study (CHARLS). Glycemic status was classified based on the 2024 American Diabetes Association (ADA) criteria (21). We aimed to systematically evaluate the association between TyG-WC and incident CVD and to be the first to compare this association across NGR, pre-DM, and DM subgroups in a Chinese population. This study seeks to fill the current evidence gap in Asian populations and to provide a scientific foundation for glycemia-based risk stratification in CVD prevention.

## 2 Materials and methods

### 2.1 Study population

This study was based on data from the China Health and Retirement Longitudinal Study (CHARLS), a nationally representative cohort that began baseline assessments in 2011. The survey covered 150 counties/districts and 450 villages/resident committees across China, encompassing 10,257 households and 17,708 respondents. Follow-up surveys were conducted in 2013, 2015, 2018, and 2020 (22). The study protocol was approved by the Biomedical Ethics Committee of Peking University (IRB00001052-11015), and all participants provided written informed consent.

**Figure 1:**
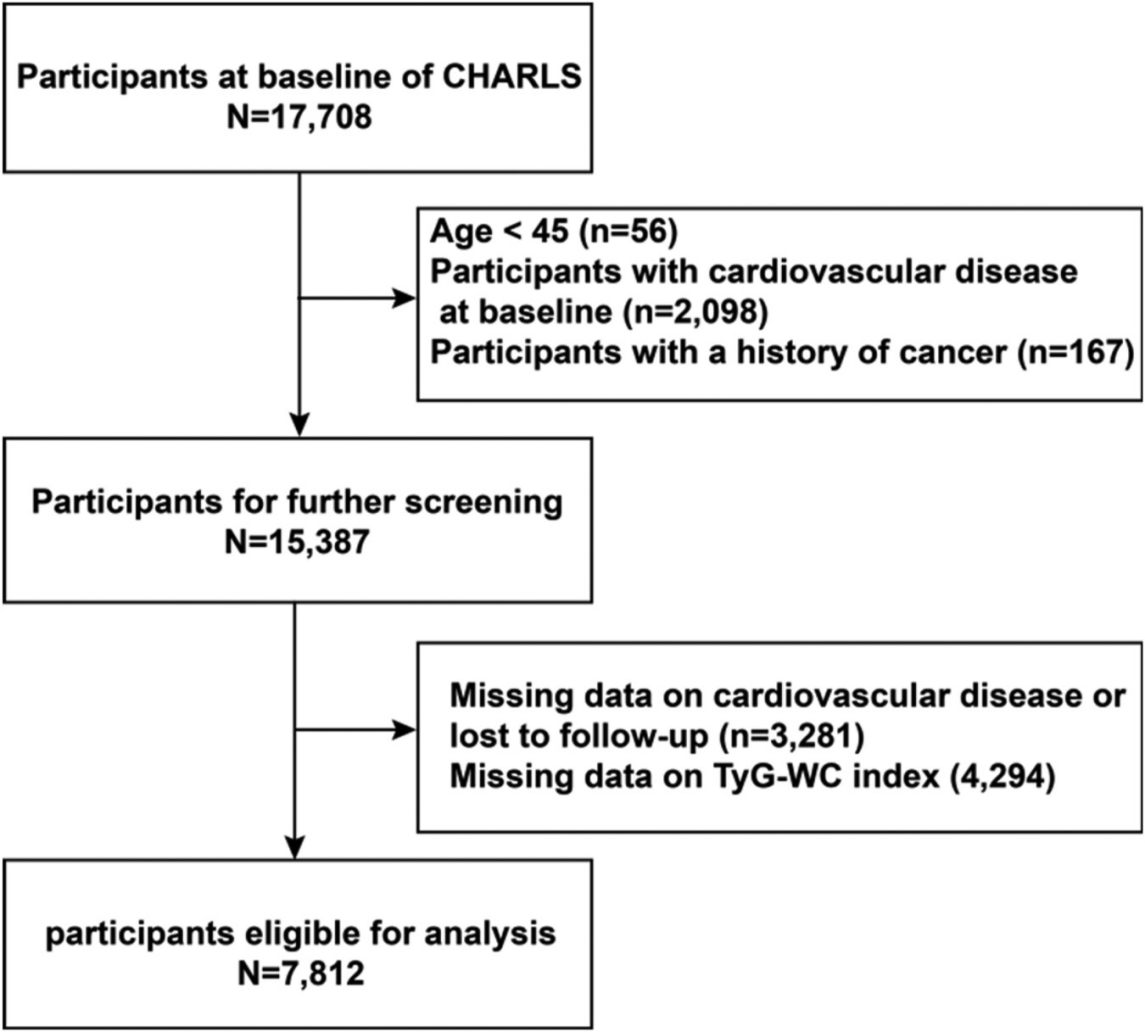
Research and design flow chart.

A total of 17,708 participants were initially enrolled in the study. The exclusion criteria were as follows: (1) Age <45 years or missing age data (*n* = 56); (2) Diagnosed with CVD at baseline (*n* = 2,098); (3) History of cancer (*n* = 167); (4) Missing CVD-related data or who were lost to follow-up (*n* = 3,281); (5) Missing TyG-WC data (*n* = 4,294). After applying these exclusion criteria, a total of 7,812 eligible participants remained for the final analysis. Participants were subsequently stratified into quartiles based on baseline TyG-WC levels. Follow-up continued through 2020 (Figure 1).

### 2.2 Study variables

#### 2.2.1 Calculation of TyG-WC

The TyG index is calculated using triglyceride (TG) and fasting plasma glucose (FPG) levels, while the TyG-WC index is derived by multiplying the TyG index by waist circumference (WC) (15, 23, 24).

$$\begin{array}{rl} & \mathrm{TyG}\;\mathrm{index}=\mathrm{In}[\mathrm{FPG}(\mathrm{mg}/\mathrm{dL})\times \mathrm{TG}(\mathrm{mg}/\mathrm{dL})/2]\\ & \mathrm{TyG}\text{-}\mathrm{WC}=\mathrm{TyG}\;\times \;\mathrm{WC} \end{array}$$

#### 2.2.2 CVD assessment

In this study, CVD was defined as the occurrence of at least one self-reported heart or vascular event, including myocardial infarction or stroke. Heart disease was identified based on participants' responses to the question: “Have you ever been diagnosed by a doctor with a heart attack, coronary heart disease, angina, congestive heart failure, or other heart problems?” Stroke was confirmed through the question: “Have you ever been diagnosed by a doctor with a stroke?” The timing of CVD onset was determined using responses to: “When did you first learn that you had a heart attack or stroke?” and “When was your most recent heart attack or stroke?” The cohort was followed by the 2011 baseline survey, with subsequent follow-ups in 2013, 2015, 2018, and 2020. Participants were tracked until the first occurrence of stroke or heart disease, or until the end of the 2020 follow-up period, whichever came first. The CVD definition and assessment method used in this study were consistent with previous research based on the CHARLS dataset (25, 26).

#### 2.2.3 Assessment of covariates

Demographic characteristics (including age, sex, marital status, education, and place of residence) and health-related information (smoking status, alcohol consumption, comorbidities, and medication use) were collected through standardized questionnaires administered by professionally trained interviewers via in-person interviews. For anthropometric measurements, height and weight were obtained using calibrated stadiometers and electronic scales with precisions of 0.1 cm and 0.1 kg, respectively. Body mass index (BMI) was calculated as weight in kilograms divided by height in meters squared (kg/m^2^). WC was measured with a non-elastic tape to the nearest 0.1 cm to ensure consistency and accuracy. Blood pressure (BP) was measured on the left arm using a validated electronic sphygmomanometer after participants had rested in a seated position for at least 15 min. Three BP measurements were taken at 45-s intervals, and the average of the three readings was used for analysis.

For biochemical assessment, all participants were instructed to fast for at least 8 h prior to blood sample collection. Blood specimens were collected and analyzed regardless of fasting status; however, data indicated that more than 92% of participants were fasting. Laboratory analyses included the following biomarkers: FPG, total cholesterol (TC), TG, high-density lipoprotein cholesterol (HDL-C), low-density lipoprotein cholesterol (LDL-C), hemoglobin A1c (HbA1c), blood urea nitrogen (BUN), and uric acid (UA).

#### 2.2.4 Definitions

The stratified analysis based on glycemic status was pre-specified in the study protocol. Glycemic status was classified according to the diagnostic criteria of the ADA (21). DM was defined as an FPG level ≥126 mg/dl, HbA1c ≥6.5%, self-reported physician diagnosis of diabetes, or current use of antidiabetic medications. Pre-DM was defined as an FPG level between 100 and 125 mg/dl or an HbA1c level between 5.7% and 6.4%. Individuals who met neither criterion were classified as having NGR. Hypertension was defined as systolic blood pressure (SBP) ≥140 mmHg and/or diastolic blood pressure (DBP) ≥90 mmHg, or current use of antihypertensive medications. Dyslipidemia was defined as meeting one or more of the following criteria: TC ≥240 mg/dl, TG ≥150 mg/dl, LDL-c ≥160 mg/dl, or HDL-c <40 mg/dl. Individuals who were taking lipid-lowering medications or had a prior diagnosis of dyslipidemia were also classified as having dyslipidemia.

### 2.3 Statistical analysis

Continuous variables with a normal distribution were presented as means (standard deviations, SD) and compared across groups using one-way analysis of variance (ANOVA). Skewed continuous variables were expressed as medians (interquartile ranges, IQRs) and assessed using the Kruskal–Wallis test. Categorical variables were reported as frequencies (percentages) and compared using the chi-square test. For missing covariate data (Supplementary Material Table 1), a missing-at-random (MAR) mechanism was assumed, and multiple imputation was applied to handle missing values. Following previous studies (27, 28), participants were categorized into four groups based on TyG-WC quartiles. Kaplan–Meier survival analysis was used to estimate the cumulative incidence of CVD across quartiles, with group differences assessed using the log-rank test. Cox proportional hazards models were fitted to examine the association between baseline TyG-WC levels and CVD risk, with results reported as HR and 95% CI. The lowest TyG-WC quartile (Q1) was used as the reference group. To explore potential nonlinear associations, restricted cubic spline (RCS) regression was applied based on multivariable-adjusted Cox models. In the diabetic subgroup, a piecewise Cox model was constructed to further assess nonlinearity, with the optimal inflection point identified using the log-likelihood ratio test. Subgroup analyses were conducted to evaluate potential effect modification by age, sex, BMI, residence, smoking status, alcohol consumption, and hypertension.

All statistical analyses were performed using R software (version 4.3.2) and EmpowerStats. All tests were two-sided, and a *P*-value < 0.05 was considered statistically significant.

## 3 Results

### 3.1 General characteristics of participants

Table 1 presents the baseline characteristics of the study population stratified by TyG-WC quartiles. The analysis revealed that as TyG-WC levels increased, the proportions of women, married individuals, those with a high school education or above, urban residents, participants without a smoking history, and those with a history of alcohol consumption progressively increased. Additionally, the prevalence of hypertension, diabetes, and dyslipidemia significantly increased with higher TyG-WC levels.

**Table 1:** Baseline characteristics of participants categorized by TyG-WC quartiles.

Characteristics	Total	Q1 ≤651.93	Q2 651.93–723.08	Q3 723.08–811.63	Q4 >811.63	*P* value
N	7,812	1,953	1,953	1,953	1,953	
Age (years)	65.91 (9.67)	66.41 (10.06)	65.85 (9.84)	65.67 (9.67)	65.70 (9.09)	0.062
Sex (%)						**<0**.**001**
Male	3,680 (47.11)	1,000 (51.20)	962 (49.26)	856 (43.83)	862 (44.14)	
Female	4,132 (52.89)	953 (48.80)	991 (50.74)	1,091 (55.86)	1,097 (56.17)	
Education (%)						**<0**.**001**
Primary school	2,286 (29.26)	635 (32.51)	578 (29.60)	547 (28.01)	526 (26.93)	
Middle school	3,222 (41.24)	818 (41.88)	818 (41.88)	814 (41.68)	772 (39.53)	
High school and above	2,304 (29.49)	500 (25.60)	557 (28.52)	592 (30.31)	655 (33.54)	
Marital status (%)						**0**.**004**
Married	6,876 (88.02)	1,677 (85.87)	1,719 (88.02)	1,734 (88.79)	1,746 (89.40)	
Unmarried	936 (11.98)	276 (14.13)	234 (11.98)	219 (11.21)	207 (10.60)	
SBP (mmHg)	129.85 (21.25)	124.30 (20.42)	127.24 (20.66)	130.98 (20.55)	136.86 (21.28)	**<0**.**001**
DBP (mmHg)	75.48 (12.05)	72.00 (11.45)	74.21 (11.92)	76.01 (11.56)	79.71 (11.92)	**<0**.**001**
BMI (kg/m2)	23.05 (20.76, 25.66)	20.11 (18.68, 21.72)	22.06 (20.56, 23.49)	24.02 (22.41, 25.64)	26.81 (24.80, 28.79)	**<0**.**001**
WC (cm)	84.13 (11.53)	71.53 (10.71)	81.13 (4.41)	87.60 (4.82)	96.25 (6.88)	**<0**.**001**
Social activities (%)						**<0**.**001**
Yes	3,879 (49.65)	1,042 (53.35)	1,031 (52.79)	932 (47.72)	874 (44.75)	
No	3,933 (50.35)	911 (46.65)	922 (47.21)	1,021 (52.28)	1,079 (55.25)	
Residence (%)						**<0**.**001**
Urban	537 (6.87)	99 (5.07)	123 (6.30)	145 (7.42)	170 (8.70)	
Rural	7,275 (93.13)	1,854 (94.93)	1,830 (93.70)	1,808 (92.58)	1,783 (91.30)	
HbAlc (%)	5.10 (4.90, 5.40)	5.00 (4.80, 5.30)	5.10 (4.80, 5.40)	5.10 (4.90, 5.40)	5.30 (5.00, 5.70)	**<0**.**001**
FPG (mg/dl)	102.24 (94.14, 113.04)	97.20 (90.18, 105.48)	100.26 (93.06, 108.18)	102.96 (95.40, 113.58)	110.52 (100.62, 130.14)	**<0**.**001**
TC (mg/dl)	193.26 (38.91)	183.18 (35.66)	190.07 (35.89)	195.05 (36.99)	204.73 (43.40)	**<0**.**001**
TG (mg/dl)	103.54 (73.46, 151.34)	69.92 (55.76, 92.04)	90.27 (70.80, 119.47)	115.05 (86.73, 153.10)	171.69 (123.01, 250.46)	**<0**.**001**
HDL-C (mg/dl)	51.39 (15.30)	59.69 (15.75)	54.93 (14.40)	49.10 (12.91)	41.83 (11.67)	**<0**.**001**
LDL-C (mg/dl)	116.32 (35.37)	109.61 (31.03)	116.55 (32.05)	120.75 (33.95)	118.37 (42.37)	**<0**.**001**
BUN (mg/dl)	15.15 (12.55, 18.23)	15.63 (12.77, 18.77)	15.18 (12.55, 18.35)	14.99 (12.38, 17.95)	14.93 (12.55, 17.87)	**<0**.**001**
UA (mg/dl)	4.45 (1.26)	4.22 (1.17)	4.30 (1.19)	4.46 (1.25)	4.81 (1.33)	**<0**.**001**
Drinking history (%)						**0**.**007**
Yes	2,674 (34.23)	720 (36.87)	683 (34.97)	624 (31.95)	647 (33.13)	
No	5,138 (65.77)	1,233 (63.13)	1,270 (65.03)	1,329 (68.05)	1,306 (66.87)	
Smoking history (%)						**<0**.**001**
Yes	3,075 (39.36)	863 (44.19)	797 (40.81)	713 (36.51)	702 (35.94)	
No	4,737 (60.64)	1,090 (55.81)	1,156 (59.19)	1,240 (63.49)	1,251 (64.06)	
History of comorbidities (%)						
Hypertension						**<0**.**001**
Yes	2,999 (38.39)	493 (25.24)	611 (31.29)	784 (40.14)	1,111 (56.89)	
No	4,813 (61.61)	1,460 (74.76)	1,342 (68.71)	1,169 (59.86)	842 (43.11)	
Diabetes						**<0**.**001**
Yes	1,157 (14.81)	113 (5.79)	183 (9.37)	272 (13.93)	589 (30.16)	
No	6,655 (85.19)	1,840 (94.21)	1,770 (90.63)	1,681 (86.07)	1,364 (69.84)	
Dyslipidaemia						**<0**.**001**
Yes	2,109 (27.00)	324 (16.59)	362 (18.54)	566 (28.98)	857 (43.88)	
No	5,703 (73.00)	1,629 (83.41)	1,591 (81.46)	1,387 (71.02)	1,096 (56.12)	
Kidney disease						0.471
Yes	1,096 (14.03)	273 (13.98)	255 (13.06)	280 (14.34)	288 (14.75)	
No	6,716 (85.97)	1,680 (86.02)	1,698 (86.94)	1,673 (85.66)	1,665 (85.25)	
History of medication use (%)						
Hypertension medications						**<0**.**001**
Yes	1,667 (21.34)	212 (10.86)	297 (15.21)	430 (22.02)	728 (37.28)	
No	6,145 (78.66)	1,741 (89.14)	1,656 (84.79)	1,523 (77.98)	1,225 (62.72)	
Diabetes medications						**<0**.**001**
Yes	239 (3.06)	15 (0.77)	31 (1.59)	46 (2.36)	147 (7.53)	
No	7,573 (96.94)	1,938 (99.23)	1,922 (98.41)	1,907 (97.64)	1,806 (92.47)	
Dyslipidemia medications						**<0**.**001**
Yes	286 (3.66)	23 (1.18)	32 (1.64)	75 (3.84)	156 (7.99)	
No	7,526 (96.34)	1,930 (98.82)	1,921 (98.36)	1,878 (96.16)	1,797 (92.01)	
GMS (%)						**<0**.**001**
NGR	3,221 (41.23)	1,132 (57.96)	927 (47.47)	735 (37.63)	427 (21.86)	
Pre-DM	3,434 (43.96)	708 (36.25)	843 (43.16)	937 (47.98)	946 (48.44)	
DM	1,157 (14.81)	113 (5.79)	183 (9.37)	272 (13.93)	589 (30.16)	
TyG-WC index						

BMI, body mass index; SBP, systolic blood pressure; DBP, diastolic blood pressure; WC, waist circumference; HbA1c, glycated hemoglobin; FPG, fasting plasma glucose; TC, total cholesterol; TG, triglyceride; HDL-C, high-density lipoprotein cholesterol; LDL-C, low-density lipoprotein cholesterol; BUN, blood urea nitrogen; UA, uric acid; GMS, glucose metabolic states; NGR, normal glucose regulation; Pre-DM, prediabetes; DM, diabetes mellitus.

Note: Bold values indicate *P* < 0.001.

Regarding laboratory biomarkers, higher TyG-WC levels were associated with an increasing trend in WC, BMI, SBP, DBP, FPG, HbA1c, TC, TG, LDL-C, and UA. Conversely, BUN and HDL-C levels decreased as TyG-WC levels increased. The baseline characteristics of participants with different glycemic statuses are presented in the supplementary tables (Supplementary Material Tables S3–S5).

### 3.2 Predictive value of baseline TyG-WC for incident CVD

During an average follow-up of 8.25 years, 1,638 participants (20.97%) experienced a first CVD event. Based on the TyG-WC quartiles (Q1–Q4), the corresponding incidence rates of CVD were 10.59, 11.29, 14.32, and 18.38 per 1,000 person-years, respectively. Kaplan–Meier cumulative incidence curves indicated a progressive increase in CVD incidence across quartiles, with statistically significant differences observed among groups (log-rank test, *P* < 0.001). Using Cox proportional hazards models, the association between baseline TyG-WC and incident CVD risk was evaluated. After adjustment for potential confounders, each 20-unit increment in TyG-WC was associated with a 3% increase in CVD risk (*HR* = 1.03, 95% *CI*: 1.02–1.04). A dose-response pattern was observed across quartiles, with higher TyG-WC quartiles showing elevated CVD risk (Q2: *HR* = 1.25, 95% *CI*: 1.05–1.37; Q3: *HR* = 1.30, 95% *CI*: 1.14–1.49; Q4: *HR* = 1.54, 95% *CI*: 1.34–1.77) (Table 2). Multivariable-adjusted RCS analysis demonstrated a significant linear dose-response association between TyG-WC and CVD risk, with no evidence of nonlinearity (*P* for overall  < 0.001; *P* for non-linear = 0.770).

### 3.3 Association between TyG-WC and CVD across different glucose metabolism statuses

**Figure 2:**
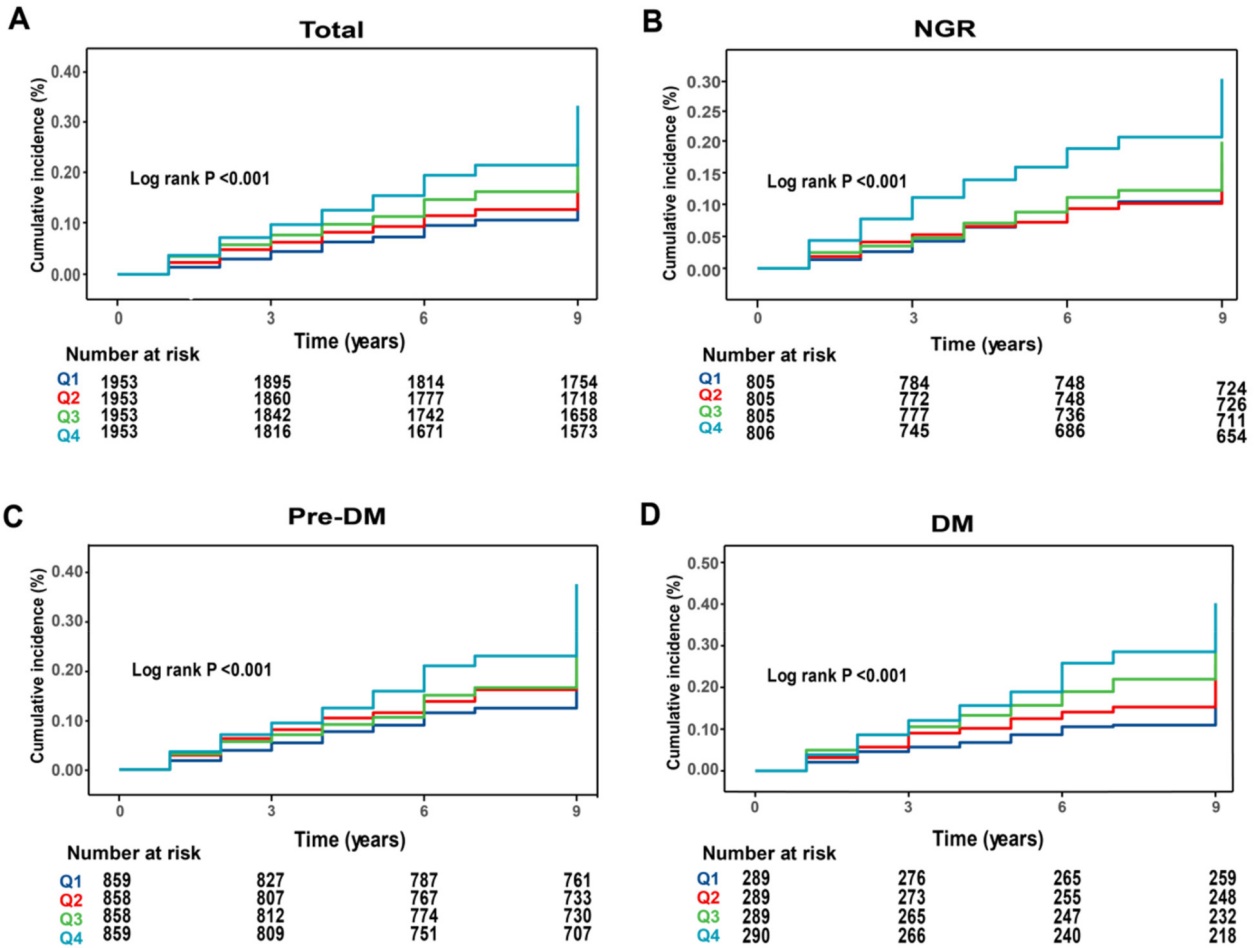
Kaplan–Meier analysis of cumulative incidence of CVD events by TyG-WC quartiles. **(A)** All participants; **(B)** NGR participants; **(C)** pre-DM participants; **(D)** DM participants.

During the follow-up period, incident CVD developed in 605 individuals with NGR (18.78%), 747 with Pre-DM (21.76%), and 286 with DM (24.72%). Kaplan–Meier survival curves (Figures 2B–D) showed that in all three glucose metabolism groups, higher TyG-WC levels were associated with a significantly increased cumulative incidence of CVD. After multivariable adjustment, compared with the lowest quartile (Q1), participants in higher TyG-WC quartiles (Q2–Q4) exhibited a significantly increased risk of incident CVD across all glucose metabolism statuses. Specifically, in the NGR group, the HRs for Q2, Q3, and Q4 were 1.16 (95% *CI*: 0.96–1.40), 1.26 (95% *CI*: 1.03–1.54), and 1.39 (95% *CI*: 1.10–1.76), respectively. In the Pre-DM group, the HRs for Q2, Q3, and Q4 were 1.25 (95% *CI*: 1.01–1.53), 1.32 (95% *CI*: 1.08–1.62), and 1.57 (95% *CI*: 1.28–1.92), respectively. In the DM group, the HRs for Q2, Q3, and Q4 were 1.93 (95% *CI*: 1.02–3.67), 2.37 (95% *CI*: 1.28–4.36), and 3.01 (95% *CI*: 1.66–5.46), respectively (Table 3).

**Figure 3:**
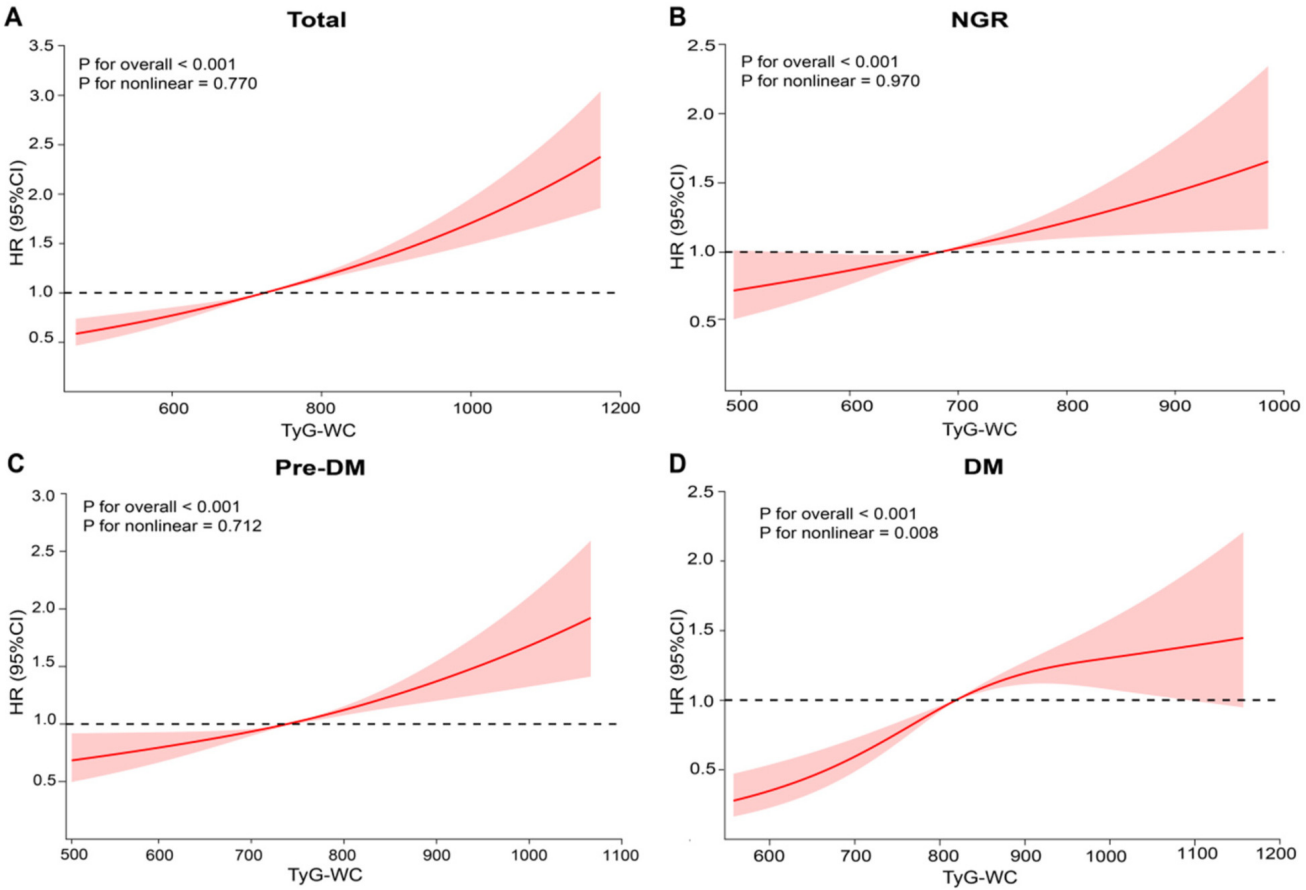
Association between baseline TyG-WC levels and CVD. **(A)** All participants; **(B)** NGR participants; **(C)** pre-DM participants; **(D)** DM participants. Adjusted for age, sex, education level, marital status, SBP, DBP, BMI, social activity, residence, HbA1c, HDL-C, LDL-C, BUN, UA, drinking history, smoking history, dyslipidemia, kidney disease, and history of medication use.

RCS analysis further confirmed these findings by demonstrating a linear positive association between baseline TyG-WC levels and incident CVD risk in both the NGR (*P* for non-linear = 0.970) and Pre-DM (*P* for non-linear = 0.712) groups (Figures 3B,C). In contrast, a non-linear association was observed in the DM group (*P* for non-linear = 0.008) (Figure 3D). Specifically, when TyG-WC was below 816.16, each 20-unit increment corresponded to a 10% increase in CVD risk (*HR* = 1.10, 95% *CI*: 1.04–1.13). However, above this threshold, the risk increase plateaued, indicating a potential saturation effect at higher TyG-WC levels (Table 4).

**Table 2:** Association between TyG-WC and CVD.

Categories	Event, *n* (%)	Model 1 *HR* (95% *CI*) *P* value	Model 2 *HR* (95% *CI*) *P* value	Model 3 *HR* (95% *CI*) *P* value
TyG-WC (per 20 units)	1,638 (20.97)	1.04 (1.03, 1.05) <0.001	1.04 (1.03, 1.05) <0.001	1.03 (1.02, 1.04) <0.001
TyG-WC quartile				
Q1	307 (15.72)	Ref	Ref	Ref
Q2	346 (17.72)	1.24 (1.09, 1.42) 0.001	1.25 (1.10, 1.43) 0.001	1.25 (1.05, 1.37) 0.007
Q3	430 (22.02)	1.40 (1.23, 1.60) <0.001	1.41 (1.23, 1.61) <0.001	1.30 (1.14, 1.49) <0.001
Q4	555 (28.42)	1.75 (1.54, 1.99) <0.001	1.76 (1.55, 2.01) <0.001	1.54 (1.34, 1.77) <0.001

Model 1: unadjusted.

Model 2: adjusted for age, sex, education level, and marital status.

Model 3: adjusted for age, sex, education level, marital status, SBP, DBP, BMI, social activity, residence, HbA1c, HDL-C, LDL-C, BUN, UA, drinking history, smoking history, diabetes, dyslipidemia, kidney disease, and history of medication use.

**Table 3:** Association between TyG-WC and CVD risk across different glucose metabolism statuses.

Categories	Event, *n* (%)	Model 1 *HR* (95% *CI*) *P* value	Model 2 *HR* (95% *CI*) *P* value	Model 3 *HR* (95% *CI*) *P* value
NGR				
TyG-WC (per 20 units)	605 (18.78)	1.03 (1.02, 1.05) <0.001	1.03 (1.02, 1.05) <0.001	1.02 (1.01, 1.04) 0.003
TyG-WC quartile				
Q1	123 (15.28)	Ref	Ref	Ref
Q2	125 (15.53)	1.21 (1.01, 1.46) 0.043	1.22 (1.01, 1.47) 0.036	1.16 (0.96, 1.40) 0.121
Q3	147 (18.26)	1.36 (1.12, 1.66) 0.002	1.37 (1.13, 1.67) 0.002	1.26 (1.03, 1.54) 0.027
Q4	210 (26.09)	1.58 (1.26, 1.97) <0.001	1.57 (1.26, 1.96) <0.001	1.39 (1.10, 1.76) 0.007
Pre-DM				
TyG-WC (per 20 units)	747 (21.76)	1.035 (1.02, 1.05) <0.001	1.035 (1.02, 1.05) <0.001	1.032 (1.02, 1.05) <0.001
TyG-WC quartile				
Q1	145 (16.87)	Ref	Ref	Ref
Q2	181 (21.09)	1.25 (1.02, 1.53) 0.033	1.28 (1.04, 1.57) 0.020	1.25 (1.01, 1.53) 0.037
Q3	187 (21.80)	1.38 (1.13, 1.67) 0.001	1.39 (1.14, 1.69) 0.001	1.32 (1.08, 1.62) 0.006
Q4	234 (27.24)	1.66 (1.37, 2.01) <0.001	1.70 (1.40, 2.06) <0.001	1.57 (1.28, 1.92) <0.001
DM				
TyG-WC (per 20 units)	286 (24.72)	1.05 (1.03, 1.06) <0.001	1.04 (1.02, 1.06) <0.001	1.04 (1.02, 1.06) <0.001
TyG-WC quartile				
Q1	44 (15.22)	Ref	Ref	Ref
Q2	65 (22.49)	1.98 (1.04, 3.75) 0.037	1.99 (1.05, 3.77) 0.035	1.93 (1.02, 3.67) 0.045
Q3	81 (28.03)	2.61 (1.42, 4.79) 0.002	2.58 (1.41, 4.75) 0.002	2.37 (1.28, 4.36) 0.006
Q4	96 (33.10)	3.58 (2.00, 6.39) <0.001	3.53 (1.98, 6.32) <0.001	3.01 (1.66,5.46) <0.001

Model 1: unadjusted.

Model 2: adjusted for age, sex, education level, and marital status.

Model 3: adjusted for age, sex, education level, marital status, SBP, DBP, BMI, social activity, residence, HbA1c, HDL-C, LDL-C, BUN, UA, drinking history, smoking history, dyslipidemia, kidney disease, and history of medication use.

### 3.4 Subgroup analysis

To further investigate the association between baseline TyG-WC levels and incident CVD events, stratified subgroup analyses were conducted based on potential risk factors. As shown in Table 5, higher TyG-WC levels were significantly associated with increased CVD incidence, and this association was consistent across all subgroups stratified by age, sex, BMI, residential location, and hypertension status. Furthermore, no significant interactions were observed between TyG-WC and other variables across glucose metabolism statuses (*P* for interaction > 0.05) (Supplementary Material Tables S6–S8).

**Table 4:** Threshold effect analysis of the nonlinear relationship between TyG-WC and CVD in individuals with DM.

Variables	Model 1 *HR* (95% *CI*) *P* value	Model 2 *HR* (95% *CI*) *P* value	Model 3 *HR* (95% *CI*) *P* value
Breakpoint (K)	816.16	816.16	816.16
<816.16 (per 20 units)	1.11 (1.06, 1.17) <0.001	1.11 (1.06, 1.15) <0.001	1.10 (1.04, 1.13) <0.001
>816.16 (per 20 units)	1.02 (1.00, 1.04) 0.1096	1.02 (1.00, 1.04) 0.058	1.02 (1.00, 1.06) 0.065
Logarithmic likelihood ratio test *P*	0.005	0.013	0.046

Model 1: unadjusted.

Model 2: adjusted for age, sex, education level, and marital status.

Model 3: adjusted for age, sex, education level, marital status, SBP, DBP, BMI, social activity, residence, HbA1c, HDL-C, LDL-C, BUN, UA, drinking history, smoking history, dyslipidemia, kidney disease, and history of medication use.

**Table 5:** Subgroup and interaction analysis of the association between TyG-WC and CVD.

Subgroups	Q1	Q2	Q3	Q4	*P* for interaction
Age					0.1870
<60	Ref	1.26 (0.94, 1.67)	1.30 (0.97, 1.75)	1.09 (0.78, 1.52)	
≥60	Ref	1.16 (1.00, 1.36)	1.20 (1.02, 1.41)	1.35 (1.13, 1.61)	
Gender					0.2114
Male	Ref	1.02 (0.84, 1.25)	1.10 (0.89, 1.35)	1.14 (0.91, 1.43)	
Female	Ref	1.34 (1.12, 1.62)	1.36 (1.12, 1.64)	1.48 (1.20, 1.81)	
residence					0.2186
Rural	Ref	1.71 (0.92, 3.18)	1.11 (0.58, 2.14)	1.50 (0.78, 2.88)	
Urban	Ref	1.16 (1.01, 1.34)	1.24 (1.08, 1.43)	1.30 (1.11, 1.51)	
BMI					0.6577
<24	Ref	0.89 (0.55, 1.43)	1.14 (0.75, 1.74)	1.50 (0.99, 2.26)	
≥24	Ref	1.15 (0.97, 1.36)	1.35 (1.13, 1.63)	1.60 (1.24, 2.05)	
Smoking history					0.1246
Yes	Ref	1.37 (1.14, 1.64)	1.34 (1.12, 1.61)	1.45 (1.19, 1.76)	
No	Ref	0.98 (0.80, 1.21)	1.12 (0.90, 1.39)	1.15 (0.90, 1.46)	
Drinking history					0.3549
Yes	Ref	1.15 (0.91, 1.46)	1.17 (0.91, 1.51)	1.08 (0.82, 1.43)	
No	Ref	1.20 (1.02, 1.42)	1.25 (1.06, 1.47)	1.39 (1.16, 1.67)	
Hypertension					
Yes	Ref	1.24 (1.04, 1.47)	1.22 (1.01, 1.46)	1.29 (1.04, 1.59)	0.8172
No	Ref	1.08 (0.87, 1.36)	1.17 (0.94, 1.46)	1.23 (0.99, 1.55)	

Adjusted for age, sex, education level, marital status, SBP, DBP, BMI, social activity, residence, HbA1c, HDL-C, LDL-C, BUN, UA, drinking history, smoking history, diabetes, dyslipidemia, kidney disease, and history of medication use.

### 3.5 Sensitivity analysis

To assess the robustness of the study findings, several sensitivity analyses were conducted. First, after excluding 665 participants who failed to fast for at least 8 h before blood sampling, the results from the Cox regression analysis remained consistent with the primary analysis (Supplementary Material Tables S9, S10). Furthermore, after excluding participants with missing data, the association between baseline TyG-WC index and CVD risk showed no significant change (Supplementary Material Tables S11, S12).

## 4 Discussion

This prospective cohort study of middle-aged and older Chinese adults shows that a higher baseline TyG-WC is significantly associated with an increased risk of incident CVD. In participants with NGR and pre-DM, TyG-WC exhibits a clear, linear, positive relationship with CVD risk; however, in individuals with diabetes, this association reaches a plateau. These findings highlight the potential importance of lowering TyG-WC as a primary prevention strategy for CVD, especially in populations with impaired glucose metabolism.

Previous studies have shown that both the TyG and WC independently predict the risk of CVD (29, 30). The combined metric, TyG-WC, provides a more comprehensive assessment of metabolic-related cardiovascular risk (31, 32). Using nine years of follow-up data from CHARLS, this study further confirmed that higher TyG-WC levels are positively associated with incident CVD. This provides longitudinal evidence for its predictive value in a Chinese population. The exact mechanisms linking TyG-WC to CVD are not fully elucidated, but IR is considered a pivotal intermediary (33). IR can promote atherosclerosis via oxidative stress, chronic low-grade inflammation, and dysregulated lipid metabolism (34); simultaneously, the accumulation of advanced glycation end products (AGEs), suppression of nitric oxide (NO) synthesis, and increased reactive oxygen species (ROS) production exacerbate endothelial injury (35, 36). IR-induced platelet activation and up-regulation of adhesion molecules further facilitate thrombosis, thereby increasing the risk of stroke and myocardial infarction (37, 38). Importantly, IR rarely occurs in isolation; rather, it interacts with multiple metabolic abnormalities, with central obesity exerting a particularly pronounced effect (39). Excess abdominal fat may trigger IR through imbalanced adipokine secretion, activation of inflammatory pathways, and impairment of insulin signaling cascades (40). Concurrent loss of skeletal muscle mass restricts glucose uptake and utilization, further reducing insulin sensitivity (41). Weight-loss interventions—especially bariatric surgery—have been shown to improve IR and related metabolic disturbances, an effect partly attributed to postoperative increases in gut hormones such as GLP-1 and PYY, which enhance insulin sensitivity and glycemic control (42). Furthermore, IR is often accompanied by central obesity (increased WC), diabetes, dyslipidemia, and hypertension, which are well-established CVD risk factors (43, 44). These factors may further explain the strong association between TyG-WC and CVD.

Furthermore, the study indicates that glucose metabolic status may modify the association between TyG-WC and CVD. Specifically, among individuals with NGR or pre-DM, TyG-WC shows a clear linear, positive relationship with CVD risk. This pattern likely reflects the ability of TyG-WC to sensitively capture latent, subclinical insulin resistance IR when the metabolic derangement is still mild (45). Unlike patients with DM, individuals with NGR and pre-DM typically receive no glucose-lowering, lipid-modifying, or antihypertensive therapy (46). Therefore, TyG-WC more accurately mirrors their degree of IR and exhibits a classic dose-response gradient. In contrast, the linear trend is absent in the diabetic subgroup. Our prospective data from a Chinese cohort reveal a nonlinear association: when TyG-WC <816, each 20-unit increment confers roughly a 10% increase in CVD risk, whereas above 816, the incremental risk plateaus. This pattern is broadly consistent with findings from U.S. populations. Zheng et al. reported that, in patients with diabetes, TyG-WC <790 or >872 was associated with 46% and 15% higher CVD risk, respectively, per 50-unit increase, while the association was flat between 790 and 872 (47). Our results corroborate this nonlinear relationship. The observed “plateau effect” may indicate saturation of vascular and metabolic injury caused by chronic glucotoxicity and lipotoxicity (48). Persistent hyperglycemia and dyslipidemia can exacerbate IR and impair endothelial function, diminishing the marginal pathogenic impact of further increases in TyG-WC (49). Ethnic and lifestyle factors may also influence the threshold at which risk saturates. For example, high-carbohydrate dietary patterns common in Chinese populations could heighten insulin demand, magnifying the adverse effect of TyG-WC on CVD. Collectively, these findings suggest the existence of a critical risk-prediction threshold for TyG-WC in diabetic patients. This hypothesis, however, warrants confirmation in large, multi-center prospective studies.

## 5 Study strengths and limitations

The primary strength of this study lies in its prospective cohort design with a 9-year follow-up, which enables a robust assessment of the long-term impact of TyG-WC on CVD risk and enhances causal inference. Additionally, this study systematically explored the differential effects of TyG-WC across various glucose metabolism statuses, providing novel insights into personalized CVD prevention strategies. Another major strength is the comprehensive definition of CVD events, encompassing myocardial infarction, angina, peripheral artery disease, and stroke, thereby improving the generalizability of the findings. Moreover, since TyG and WC are routinely measured in clinical practice, the TyG-WC index can be easily calculated from these parameters, making it a simple, cost-effective, and practical tool for CVD risk assessment. Given these advantages, the use of TyG-WC as an efficient and convenient marker for CVD risk prediction is both scientifically justified and clinically feasible.

Despite the significance of this study, several limitations should be acknowledged. First, although multiple confounding factors were adjusted for in the statistical analyses, the influence of unmeasured or unknown confounders—such as genetic background, dietary patterns, and long-term lifestyle changes—cannot be entirely ruled out. These factors may have played a role in the observed association between the TyG-WC and CVD. Second, although glycemic status was categorized using established diagnostic criteria, individuals with borderline glucose levels may have been misclassified. Such misclassification could have led to erroneous group assignments, thereby affecting the precision of the association between TyG-WC and CVD. Third, the CHARLS database does not systematically collect information on certain clinical cardiovascular conditions, such as cardiomyopathy, congenital vascular malformations, atrial fibrillation, and carotid artery stenosis. Although the overall prevalence of these conditions is relatively low in middle-aged and older populations, they are important risk factors for CVD, and failure to account for them may result in residual confounding. Additionally, CVD outcomes in this study were identified based on self-reported physician diagnoses. While this approach has been widely adopted in previous CHARLS-based studies, it remains susceptible to recall bias and misclassification. Furthermore, due to the lack of specific biomarkers in the CHARLS database (e.g., C-peptide levels or islet autoantibodies), this study could not definitively distinguish and exclude individuals with type 1 diabetes. Although type 2 diabetes accounts for the vast majority of diabetes cases in this population, a small degree of misclassification may exist. This should be considered when interpreting the findings. Lastly, this study assessed the association between TyG-WC and CVD risk based solely on baseline measurements. The potential impact of longitudinal changes or trends in TyG-WC over the follow-up period was not evaluated. Future studies should explore the trajectories of TyG-WC over time to more comprehensively assess its predictive value in CVD prevention.

## 6 Conclusion

This study found that higher TyG-WC levels were significantly associated with an increased risk of CVD, showing a linear relationship among individuals with normal glucose regulation and prediabetes, while the association plateaued in those with diabetes. TyG-WC may serve as an effective biomarker for the early identification of high-risk individuals, providing valuable evidence for preventive screening and personalized health management.

## Data Availability

The original contributions presented in the study are included in the article/Supplementary Material, further inquiries can be directed to the corresponding author.
