# Supplementary material for: Association between the triglyceride glucose-waist circumference index and cardiovascular disease across different glycemic statuses among middle-aged and older Chinese adults
Source: Front Cardiovasc Med. 2025 Jul 14;12:1608655. doi: 10.3389/fcvm.2025.1608655 (PMC12301363; doi:10.3389/fcvm.2025.1608655)
Supplement: Supplementary file 1 [file Datasheet1.pdf]

Supplementary Material

- **Table S1** Distribution of missing data.
- **Table S2** Collinearity diagnostic steps for TyG-WC with other covariates.
- **Table S3** Baseline characteristics of participants with NGR.
- **Table S4** Baseline characteristics of participants with Pre-DM.
- **Table S5** Baseline characteristics of participants with DM.
- **Table S6** Subgroup and interaction analysis of the association between TyG-WC and CVD in the NGR population.
- **Table S7** Subgroup and interaction analysis of the association between TyG-WC and CVD in the Pre-DM population.
- **Table S8** Subgroup and interaction analysis of the association between TyG-WC and CVD in the DM population.
- **Table S9** The association between TyG-WC and CVD after excluding individuals with less than 8 hours of fasting.
- **Table S10** The association between TyG-WC and CVD according to glucose metabolic states after excluding individuals with less than 8 hours of fasting.
- **Table S11** Association between TyG-WC and CVD after excluding individuals with missing covariates.
- **Table S12** Association between TyG-WC and the risk of CVD according to glucose

metabolic states after excluding individuals with missing covariates.

Table S1 Distribution of missing data.

Characteristics	No. of missing values	Percent(%)	Disposition
Age	0	0	-
Sex	0	0	-
Education	0	0	-
Marital status	0	0	-
SBP	97	1.24	Multiple imputation
DBP	98	1.25	Multiple imputation
BMI	107	1.36	Multiple imputation
WC	0	0	
Social activities	0	0	
Residence	2	0.03	Multiple imputation
HbA1c	62	0.79	Multiple imputation
FPG	0	0	
TC	0	0	
TG	0	0	
HDL-C	1	0.01	Multiple imputation
LDL-C	15	0.19	Multiple imputation
BUN	2	0.03	Multiple imputation
UA	0	0	Multiple imputation
Smoking	9	0.12	Multiple imputation
Drinking	8	0.10	Multiple imputation
Hypertension	0	0	-
DM	0	0	-
Dyslipidemia	0	0	-
Kidney disease	0	0	-
Hypertension medications	0	0	-
Diabetes medications	0	0	-
Dyslipidemia medications	0	0	-
TyG-WC	0	0	-

Abbreviations: SBP, Systolic blood pressure; DBP, Diastolic blood pressure; BMI, Body mass index; HbA1c, Hemoglobin A1c; FPG, Fasting plasma glucose; TC, Total cholesterol; TG, Triglyceride; HDL-C, High-density lipoprotein cholesterol; LDL-C, Low-density lipoprotein cholesterol; BNU, blood urea nitrogen; UA, uric acid; DM, diabetes mellitus; TyG-WC, triglyceride glucose waist circumference.

Table S2 Collinearity diagnostic steps for TyG-WC with other covariates.

Variable	Step1	Step2	Step3
TyG-WC	40.9	2.5	2.5
Age	1.7	1.7	1.7
Sex	2.6	2.6	2.6
Education	1.4	1.4	1.4
Marital status	1.1	1.1	1.1
SBP	3.4	3.4	3.4
DBP	2.6	2.6	2.6
BMI	1.9	1.9	1.9
WC	28	NA	NA
Social activities	1	1	1
Residence	1.1	1.1	1.1
HbA1c	1.9	1.9	1.9
FPG	3.1	2.5	2.5
TC	16	16	NA
TG	11.1	6.8	1.8
HDL-C	3.6	3.6	1.4
LDL-C	13.1	13	1.1
BUN	1.1	1.1	1.1
UA	1.4	1.3	1.3
Smoking	2	2	2
Drinking	1.4	1.4	1.4
Hypertension	3.1	3.1	3.1
Diabetes	1.9	1.8	1.8
Dyslipidemia	1.2	1.2	1.2
Kidney disease	1	1	1
Hypertension medications	1.9	1.9	1.9
Diabetes medications	1.3	1.3	1.3
Dyslipidemia medications	1.2	1.2	1.2

Abbreviations: TyG-WC, triglyceride glucose waist *CI*rcumference; SBP, Systolic blood pressure; DBP, Diastolic blood pressure; BMI, Body mass index; HbA1c, Hemoglobin A1c; FPG, Fasting plasma glucose; TC, Total cholesterol; TG, Triglyceride; HDL-C, High-density lipoprotein cholesterol; LDL-C, Low-density lipoprotein cholesterol; BNU, blood urea nitrogen; UA, uric acid; DM, diabetes mellitus.

Table S3 Baseline characteristics of participants with NGR

Characteristics	Total	Q1<br>≤ 631.62	Q2<br>631.62 – 671.18	Q3<br>671.18 – 811.63	Q4<br>> 717.06	*P* value
**N**	3221	805	805	805	806	
**Age (years)**	65.12 (9.88)	65.93 (10.20)	64.94 (9.90)	64.99 (9.90)	64.62 (9.47)	**0.046**
**Sex (%)**						**<0.001**
Male	1495 (46.46)	400 (49.69)	412 (51.24)	346 (42.98)	337 (41.92)	
Female	1723 (53.54)	405 (50.31)	392 (48.76)	459 (57.02)	467 (58.08)	
**Education (%)**						**<0.001**
Primary school	942 (29.25)	263 (32.67)	231 (28.70)	250 (31.06)	198 (24.57)	
Middle school	1336 (41.48)	346 (42.98)	337 (41.86)	318 (39.50)	335 (41.56)	

Characteristics	Total	Q1<br>≤ 631.62	Q2<br>631.62 – 671.18	Q3<br>671.18 – 811.63	Q4<br>> 717.06	*P* value
High school and above	943 (29.28)	196 (24.35)	237 (29.44)	237 (29.44)	273 (33.87)	
**Marital status (%)**						0.058
Married	2868 (89.04)	700 (86.96)	711 (88.32)	731 (90.81)	726 (90.07)	
Unmarried	353 (10.96)	105 (13.04)	94 (11.68)	74 (9.19)	80 (9.93)	
**SBP (mmHg)**	126.68 (20.75）	122.23 (20.23)	124.18 (19.48)	127.59 (20.56)	132.74 (21.20)	**<0.001**
**DBP (mmHg)**	74.26 (12.29）	71.18 (11.68)	73.10 (11.68)	74.90 (12.67)	77.89 (12.12)	**<0.001**
**WC (cm)**	82.52 (10.93)	70.70 (9.95)	79.49 (3.97)	85.29 (4.36)	94.61 (6.46)	**<0.001**
**Drinking history (%)**						**0.031**
Yes	1104 (34.30)	292 (36.36)	299 (37.14)	260 (32.30)	253 (31.39)	
No	2115 (65.70)	511 (63.64)	506 (62.86)	545 (67.70)	553 (68.61)	
**Smoking history (%)**						**<0.001**
Yes	1271 (39.50)	358 (44.64)	345 (42.86)	284 (35.28)	284 (35.24)	
No	1947 (60.50)	444 (55.36)	460 (57.14)	521 (64.72)	522 (64.76)	
**Social activities (%)**						**0.003**
Yes	1578 (48.99)	423 (52.55)	410 (50.93)	392 (48.70)	353 (43.80)	
No	1643 (51.01)	382 (47.45)	395 (49.07)	413 (51.30)	453 (56.20)	
**Residence (%)**						0.940
Urban	201 (6.24)	51 (6.34)	47 (5.84)	53 (6.58)	50 (6.20)	
Rural	3020 (93.76)	754 (93.66)	758 (94.16)	752 (93.42)	756 (93.80)	
**HbAlc (%)**	5.01 (0.39)	4.98 (0.38)	5.00 (0.37)	5.01 (0.42)	5.04 (0.38)	**0.016**
**FPG (mg/dl)**	90.80 (8.50)	88.92 (10.27)	90.20 (8.53)	91.46 (7.54)	92.62 (6.81)	**<0.001**
**TG (mg/dl)**	106.11 (56.46)	73.04 (28.33)	90.40 (38.05)	108.85 (42.53)	152.09 (72.34)	**<0.001**
**HDL-C (mg/dl)**	53.11 (14.47)	59.75 (15.17)	56.22 (13.69)	51.64 (13.13)	44.86 (11.18)	**<0.001**
**LDL-C (mg/dl)**	113.67 (31.23)	105.57 (29.30)	111.70 (29.26)	117.95 (30.64)	119.47 (33.62)	**<0.001**
**BMI (kg/m2)**	22.39 (20.35, 24.83)	19.80 (18.35,21.21)	21.50 (20.16,22.83)	23.19 (21.75,24.72)	26.05 (24.25,28.13)	**<0.001**
**TC (mg/dl)**	184.41 (161.99, 207.99)	175.13 (154.25,199.49)	182.86 (159.67,204.12)	187.89 (166.62,209.92)	191.75 (169.72,216.88)	**<0.001**
**BUN**	14.96 (12.35,	15.57 (12.52,18.29)	15.01 (12.55,18.12)	14.76 (12.21,17.70)	14.58 (12.16,17.34)	**0.001**

Characteristics	Total	Q1<br>≤ 631.62	Q2<br>631.62 – 671.18	Q3<br>671.18 – 811.63	Q4<br>> 717.06	*P* value
	17.90)					
**UA**	4.33 (1.20)	4.19 (1.14)	4.29 (1.18)	4.25 (1.20)	4.59 (1.22)	**<0.001**

BMI, Body Mass Index; SBP, Systolic Blood Pressure; DBP, Diastolic Blood Pressure; WC, Waist Circumference; HbA1c, Glycated Hemoglobin; FPG, Fasting Plasma Glucose; TC, Total Cholesterol; TG, Triglyceride; HDL-C, High-Density Lipoprotein Cholesterol; LDL-C, Low-Density Lipoprotein Cholesterol; BUN, Blood Urea Nitrogen; UA, Uric Acid.

Table S4 Baseline characteristics of participants with Pre-DM

Characteristics	Total	Q1<br>≤ 669.90	Q2<br>669.90 – 748.35	Q3<br>748.35 – 822.29	Q4<br>> 822.29	*P* value
**N**	3434	859	858	858	859	
**Age (years)**	66.16 (9.49)	66.92 (9.79)	66.43 (9.69)	65.76 (9.42)	65.53 (8.99)	**0.009**
**Sex (%)**						**<0.001**
Male	1617 (47.13)	454 (52.98)	422 (49.24)	363 (42.31)	378 (44.00)	
Female	1814 (52.87)	403 (47.02)	435 (50.76)	495 (57.69)	481 (56.00)	
**Education (%)**						**0.017**
Primary school	1012 (29.47)	280 (32.60)	248 (28.90)	252 (29.37)	232 (27.01)	
Middle school	1398 (40.71)	354 (41.21)	367 (42.77)	339 (39.51)	338 (39.35)	
High school and above	1024 (29.82)	225 (26.19)	243 (28.32)	267 (31.12)	289 (33.64)	
**Marital status (%)**						**0.008**
Married	3001 (87.39)	729 (84.87)	742 (86.48)	756 (88.11)	774 (90.10)	
Unmarried	433 (12.61)	130 (15.13)	116 (13.52)	102 (11.89)	85 (9.90)	
**SBP (mmHg)**	130.98 (21.30)	126.36 (21.11)	128.65 (20.33)	131.66 (20.53)	137.29 (21.63)	**<0.001**
**DBP (mmHg)**	76.04 (11.94)	72.90 (11.55)	74.85 (11.55)	76.48 (11.37)	79.95 (12.16)	**<0.001**
**WC (cm)**	84.65 (11.50)	71.81 (10.65)	81.81 (4.39)	88.20 (4.61)	96.77 (6.48)	**<0.001**
**Drinking history (%)**						**0.036**
Yes	1172 (34.17)	316 (36.83)	309 (36.10)	267 (31.16)	280 (32.60)	
No	2258 (65.83)	542 (63.17)	547 (63.90)	590 (68.84)	579 (67.40)	
**Smoking history (%)**						**<0.001**
Yes	1337 (38.98)	387 (45.10)	347 (40.54)	291 (33.96)	312 (36.32)	
No	2093	471 (54.90)	509 (59.46)	566 (66.04)	547 (63.68)	

Characteristics	Total	Q1<br>≤ 669.90	Q2<br>669.90 – 748.35	Q3<br>748.35 – 822.29	Q4<br>> 822.29	*P* value
	(61.02)					
**Social activities (%)**						**<0.001**
Yes	1755 (51.11)	492 (57.28)	460 (53.61)	413 (48.14)	390 (45.40)	
No	1679 (48.89)	367 (42.72)	398 (46.39)	445 (51.86)	469 (54.60)	
**Residence (%)**						**<0.001**
Urban	235 (6.84)	36 (4.19)	51 (5.94)	76 (8.86)	72 (8.38)	
Rural	3199 (93.16)	823 (95.81)	807 (94.06)	782 (91.14)	787 (91.62)	
**HbAlc (%)**	5.19 (0.43)	5.17 (0.42)	5.16 (0.41)	5.19 (0.41)	5.24 (0.45)	**<0.001**
**FPG (mg/dl)**	108.41 (7.24)	107.38 (7.38)	107.51 (7.01)	108.77 (6.93)	109.98 (7.33)	**<0.001**
**TG (mg/dl)**	132.67 (86.62)	83.23 (42.29)	108.28 (54.75)	136.10 (63.96)	203.04 (114.64)	**<0.001**
**HDL-C (mg/dl)**	51.44 (15.48)	60.41 (16.59)	54.52 (14.56)	48.67 (12.77)	42.15 (11.17)	**<0.001**
**LDL-C (mg/dl)**	119.54 (35.96)	113.94 (34.01)	119.40 (32.69)	123.36 (35.41)	121.46 (40.59)	**<0.001**
**BMI (kg/m2)**	23.29 (20.91, 25.90)	20.12 (18.80,21.70)	22.32 (20.73,23.69)	24.22 (22.71,25.90)	27.07 (25.33,28.84)	**<0.001**
**TC (mg/dl)**	193.69 (170.49, 219.20)	185.57 (163.15,209.15)	192.53 (170.10,216.01)	195.23 (171.65,220.75)	203.35 (177.06,229.25)	**<0.001**
**BUN**	15.21 (12.63, 18.37)	15.71 (12.83,19.02)	15.32 (12.69,18.59)	14.96 (12.49,17.93)	14.87 (12.53,17.70)	**<0.001**
**UA**	4.52 (1.26)	4.25 (1.18)	4.42 (1.22)	4.50 (1.25)	4.91 (1.30)	**<0.001**

BMI, Body Mass Index; SBP, Systolic Blood Pressure; DBP, Diastolic Blood Pressure; WC, Waist Circumference; HbA1c, Glycated Hemoglobin; FPG, Fasting Plasma Glucose; TC, Total Cholesterol; TG, Triglyceride; HDL-C, High-Density Lipoprotein Cholesterol; LDL-C, Low-Density Lipoprotein Cholesterol; BUN, Blood Urea Nitrogen; UA, Uric Acid.

Table S5 Baseline characteristics of participants with DM

Characteristics	Total	Q1<br>≤ 748.99	Q2<br>748.99 – 806.28	Q3<br>806.28 – 892.16	Q4<br>> 892.16	*P* value
**N**	1157	289	289	289	290	
**Age (years)**	67.37 (9.43)	68.48 (10.28)	67.87 (9.52)	67.38 (9.04)	65.77 (8.65)	**0.004**
**Sex (%)**						**0.002**
Male	568 (49.09)	157 (54.33)	144 (49.83)	115 (39.79)	152 (52.41)	
Female	589 (50.91)	132 (45.67)	145 (50.17)	174 (60.21)	138 (47.59)	
**Education (%)**						**<0.001**
Primary school	332 (28.69)	91 (31.49)	81 (28.03)	95 (32.87)	65 (22.41)	

Characteristics	Total	Q1<br>≤ 748.99	Q2<br>748.99 – 806.28	Q3<br>806.28 – 892.16	Q4<br>> 892.16	*P* value
Middle school	488 (42.18)	131 (45.33)	137 (47.40)	95 (32.87)	125 (43.10)	
High school and above	337 (29.13)	67 (23.18)	71 (24.57)	99 (34.26)	100 (34.48)	
**Marital status (%)**						0.090
Married	1007 (87.04)	249 (86.16)	242 (83.74)	253 (87.54)	263 (90.69)	
Unmarried	150 (12.96)	40 (13.84)	47 (16.26)	36 (12.46)	27 (9.31)	
**SBP (mmHg)**	134.46 (21.10)	129.49 (22.55)	133.62 (19.58)	134.72 (20.85)	140.02 (20.03)	**<0.001**
**DBP (mmHg)**	77.07 (11.40)	73.59 (11.00)	75.77 (10.82)	77.94 (11.45)	80.97 (11.06)	**<0.001**
**WC (cm)**	87.03 (12.48)	73.38 (13.08)	85.08 (5.04)	90.91 (5.68)	98.70 (7.29)	**<0.001**
**Drinking history (%)**						0.266
Yes	760 (65.80)	183 (63.54)	194 (67.36)	201 (69.55)	182 (62.76)	
No	395 (34.20)	105 (36.46)	94 (32.64)	88 (30.45)	108 (37.24)	
**Smoking history (%)**						**0.004**
Yes	693 (60.00)	159 (55.21)	167 (57.99)	199 (68.86)	168 (57.93)	
No	462 (40.00)	129 (44.79)	121 (42.01)	90 (31.14)	122 (42.07)	
**Social activities (%)**						0.058
Yes	546 (47.19)	151 (52.25)	139 (48.10)	137 (47.40)	119 (41.03)	
No	611 (52.81)	138 (47.75)	150 (51.90)	152 (52.60)	171 (58.97)	
**Residence (%)**						0.068
Urban	101 (8.74)	15 (5.19)	25 (8.68)	29 (10.03)	32 (11.07)	
Rural	1054 (91.26)	274 (94.81)	263 (91.32)	260 (89.97)	257 (88.93)	
**HbAlc (%)**	6.11 (1.55)	5.72 (1.39)	5.96 (1.47)	6.21 (1.61)	6.55 (1.58)	**<0.001**
**FPG (mg/dl)**	165.60 (66.47)	147.19 (41.13)	155.21 (52.18)	169.01 (76.14)	190.89 (79.97)	**<0.001**
**TG (mg/dl)**	203.07 (215.27)	97.20 (62.63)	132.54 (77.15)	203.24 (136.05)	278.70 (231.58)	**<0.001**
**HDL-C (mg/dl)**	46.46 (15.89)	57.93 (17.42)	49.09 (13.08)	43.29 (11.87)	35.57 (11.36)	**<0.001**
**LDL-C (mg/dl)**	112.67 (40.66)	113.69 (35.04)	117.50 (35.26)	115.19 (40.62)	103.96 (49.36)	**<0.001**
**BMI (kg/m2)**	23.88 (21.70, 26.72)	20.97 (19.31,22.84)	22.87 (21.30,24.51)	24.85 (23.48,27.11)	27.60 (25.42,29.51)	**<0.001**
**TC (mg/dl)**	195.62 (169.33, 225.00)	185.57 (161.99,214.18)	189.05 (167.01,213.40)	202.19 (172.04,227.32)	207.60 (178.61,243.56)	**<0.001**
**BUN**	15.43 (12.94, 18.60)	15.84 (12.67,19.33)	15.52 (13.08,18.79)	14.73 (12.41,17.87)	15.99 (13.64,18.46)	**0.014**
**UA**	4.56 (1.37)	4.21 (1.22)	4.54 (1.35)	4.55 (1.37)	4.94 (1.42)	**<0.001**

BMI, Body Mass Index; SBP, Systolic Blood Pressure; DBP, Diastolic Blood Pressure; WC, Waist Circumference;

HbA1c, Glycated Hemoglobin; FPG, Fasting Plasma Glucose; TC, Total Cholesterol; TG, Triglyceride; HDL-C, High-Density Lipoprotein Cholesterol; LDL-C, Low-Density Lipoprotein Cholesterol; BUN, Blood Urea Nitrogen; UA, Uric Acid.

Table S6 Subgroup and interaction analysis of the association between TyG-WC and CVD in the NGR population.

Subgroups	Q1	Q2	Q3	Q4	*P* for interaction
**Age**					0.6983
≤60	Ref	1.41 (0.91, 2.18)	1.27 (0.81, 1.99)	1.30 (0.82, 2.07)	
>60	Ref	0.97 (0.75, 1.26)	1.13 (0.88, 1.46)	1.21 (0.92, 1.60)	
**Sex**					0.5650
Male	Ref	1.00 (0.73, 1.37)	1.05 (0.76, 1.46)	1.13 (0.80, 1.61)	
Female	Ref	1.19 (0.87, 1.62)	1.30 (0.96, 1.75)	1.37 (0.99, 1.89)	
**Residence**					0.7304
Rural	Ref	1.21 (0.41, 3.57)	0.56 (0.17, 1.87)	0.43 (0.10, 1.92)	
Urban	Ref	1.04 (0.83, 1.31)	1.16 (0.92, 1.45)	1.21 (0.96, 1.54)	
**BMI**					0.7580
<24	Ref	0.85 (0.34, 2.13)	0.83 (0.36, 1.94)	1.25 (0.55, 2.83)	
≥24	Ref	0.90 (0.68, 1.17)	1.16 (0.88, 1.52)	1.47 (1.03, 2.11)	
**Smoking**					0.6937
Yes	Ref	1.15 (0.85, 1.55)	1.17 (0.87, 1.57)	1.24 (0.91, 1.68)	
No	Ref	1.03 (0.74, 1.43)	1.16 (0.83, 1.62)	1.20 (0.83, 1.72)	
**Drinking**					0.2619
Yes	Ref	1.12 (0.86, 1.48)	1.21 (0.93, 1.59)	1.32 (1.00, 1.75)	
No	Ref	0.98 (0.67, 1.44)	1.06 (0.71, 1.57)	1.04 (0.67, 1.61)	
**Hypertension**					
Yes	Ref	0.97 (0.73, 1.27)	1.16 (0.88, 1.54)	1.27 (0.93, 1.73)	0.1135
No	Ref	1.29 (0.88, 1.88)	1.18 (0.81, 1.71)	1.18 (0.81, 1.72)	

Adjusted for age, sex, education level, marital status, SBP, DBP, BMI, social activity, residence, HbA1c, HDL-C, LDL-C, BUN, UA, drinking history, smoking history, dyslipidemia, kidney disease, and history of medication use.

Table S7 Subgroup and interaction analysis of the association between TyG-WC and CVD in the Pre-DM population.

Subgroups	Q1	Q2	Q3	Q4	*P* for interaction
**Age**					0.1861
≤60	Ref	0.96 (0.963, 1.48)	0.96 (0.61, 1.50)	0.69 (0.41, 1.15)	
>60	Ref	1.25 (1.00, 1.55)	1.18 (0.94, 1.48)	1.45 (1.14， 1.85)	
**Sex**					0.1085
Male	Ref	1.13 (0.85, 1.51)	1.11 (0.81, 1.52)	1.33 (0.96, 1.85)	
Female	Ref	1.30 (1.00, 1.69)	1.22 (0.94, 1.60)	1.33 (0.99, 1.78)	
**Residence**					0.5208
Rural	Ref	1.63 (0.44, 6.08)	0.58 (0.13, 2.65)	0.56 (0.10, 3.35)	
Urban	Ref	1.05 (0.83, 1.33)	1.13 (0.89, 1.44)	1.25 (0.97, 1.62)	
**BMI**					0.9510
<24	Ref	1.25 (0.56, 2.79)	1.34 (0.36, 4.94)	2.25 (0.25, 9.91)	
≥24	Ref	1.24 (0.99, 1.54)	1.06 (0.82, 1.36)	1.14 (0.81, 1.60)	
**Smoking**					0.9264
Yes	Ref	1.32 (1.02, 1.70)	1.27 (0.99, 1.65)	1.42 (1.07, 1.88)	
No	Ref	1.10 (0.81, 1.49)	1.04 (0.74, 1.47)	1.21 (0.85, 1.71)	
**Drinking**					0.6984
Yes	Ref	1.19 (0.95, 1.50)	1.11 (0.87, 1.41)	1.34 (1.03, 1.73)	
No	Ref	1.22 (0.86, 1.75)	1.28 (0.87, 1.86)	1.18 (0.78, 1.78)	
**Hypertension**					0.5601
Yes	Ref	1.16 (0.90, 1.49)	1.10 (0.84, 1.44)	1.25 (0.92, 1.69)	
No	Ref	1.29 (0.94, 1.76)	1.20 (0.88, 1.65)	1.33 (0.96, 1.83)	

Adjusted for age, sex, education level, marital status, SBP, DBP, BMI, social activity, residence, HbA1c, HDL-C, LDL-C, BUN, UA, drinking history, smoking history, dyslipidemia, kidney disease, and history of medication use.

Table S8 Subgroup and interaction analysis of the association between TyG-WC and CVD in the DM population.

Subgroups	Q1	Q2	Q3	Q4	*P* for interaction
**Age**					0.0989
≤60	Ref	2.17 (0.75, 6.26)	1.94 (0.67, 5.65)	2.21 (0.65, 7.53)	

>60	Ref	1.29 (0.88, 1.88)	1.21 (0.81, 1.82)	1.36 (0.87，2.11)	
**Sex**					0.4548
Male	Ref	0.97 (0.56, 1.69)	0.92 (0.50, 1.69)	0.94 (0.45, 1.94)	
Female	Ref	1.78 (1.10, 2.88)	1.73 (1.05, 2.85)	1.96 (1.15, 3.37)	
**Residence**					0.5208
Rural	Ref	1.63 (0.44, 6.08)	0.58 (0.13, 2.65)	0.56 (0.10, 3.35)	
Urban	Ref	1.05 (0.83, 1.33)	1.13 (0.89, 1.44)	1.25 (0.97, 1.62)	
**BMI**					0.6229
<24	Ref	1.23 (0.87, 2.03)	1.12 (0.64, 1.97)	1.20 (0.56, 2.54)	
⩾24	Ref	1.48 (0.64, 3.43)	1.49 (0.66, 3.35)	1.74 (0.76, 3.98)	
**Smoking**					0.7261
Yes	Ref	1.52 (0.96, 2.42)	1.37 (0.85, 2.21)	1.70 (1.02, 2.81)	
No	Ref	1.15 (0.64, 2.05)	1.20 (0.63, 2.31)	1.06 (0.48, 2.35)	
**Drinking**					0.6984
Yes	Ref	1.73 (0.42, 1.51)	1.67 (1.06, 1.41)	1.34 (1.03, 1.73)	
No	Ref	1.22 (0.86, 1.75)	1.28 (0.87, 1.86)	1.18 (0.78, 1.78)	
**Hypertension**					0.9658
Yes	Ref	1.16 (0.69, 1.97)	1.08 (0.61, 1.92)	1.26 (0.65, 2.46)	
No	Ref	1.65 (1.00, 2.73)	1.53 (0.90, 2.58)	1.89 (1.07, 3.37)	

Adjusted for age, sex, education level, marital status, SBP, DBP, BMI, social activity, residence, HbA1c, HDL-C, LDL-C, BUN, UA, drinking history, smoking history, dyslipidemia, kidney disease, and history of medication use.

Table S9 The association between TyG-WC and CVD after excluding individuals with less than 8 hours of fasting.

Categories	Event, n (%)	Model 1 *HR* (95% *CI*) *P* value	Model 2 *HR* (95% *CI*) *P* value	Model 3 *HR* (95% *CI*) *P* value
**TyG-WC (per 20 units)**	1511 (21.14)	1.04 (1.03, 1.05) <0.001	1.04 (1.03, 1.05) <0.001	1.02 (1.01, 1.03) <0.001
**TyG-WC quartile**				
**Q1**	289 (16.17)	Ref	Ref	Ref

Q2	314 (17.58)	1.25 (1.08, 1.43) 0.002	1.25 (1.08, 1.44) 0.002	1.19 (1.03, 1.37) 0.017
Q3	379 (21.21)	1.49 (1.30, 1.71) <0.001	1.48 (1.29, 1.69) <0.001	1.28 (1.11, 1.48) <0.001
Q4	509 (28.48)	1.85 (1.62, 2.11) <0.001	1.83 (1.60, 2.09) <0.001	1.34 (1.14, 1.57) <0.001

Model 1: Unadjusted.

Model 2: Adjusted for age, sex, education level, and marital status.

Model 3: Adjusted for age, sex, education level, marital status, SBP, DBP, BMI, social activity, residence, HbA1c, HDL-C, LDL-C, BUN, UA, drinking history, smoking history, diabetes, dyslipidemia, kidney disease, and history of medication use.

Table S10 The association between TyG-WC and CVD according to glucose metabolic states after excluding individuals with less than 8 hours of fasting .

Categories	Event, n (%)	Model 1 *HR* (95% *CI*) *P* value	Model 2 *HR* (95% *CI*) *P* value	Model 3 *HR* (95% *CI*) *P* value
**NGR**				
**TyG-WC (per 20 units)**	571 (19.07)	1.04 (1.02, 1.06) <0.001	1.04 (1.02, 1.06) <0.001	1.02 (1.00, 1.04) 0.047
**TyG-WC quartile**				
**Q1**	118 (15.78)	Ref	Ref	Ref
**Q2**	119 (15.91)	1.08 (0.86, 1.36) 0.499	1.11 (0.89, 1.40) 0.354	1.09 (0.87, 1.37) 0.461
**Q3**	134 (17.91)	1.21 (0.97, 1.52) 0.087	1.24 (0.99, 1.55) 0.064	1.15 (0.91, 1.44) 0.249
**Q4**	200 (26.70)	1.59 (1.29, 1.97) <0.001	1.63 (1.31, 2.03) <0.001	1.28 (1.00, 1.63) 0.047
**Pre-DM**				
**TyG-WC (per 20 units)**	690 (21.77)	1.04 (1.02, 1.05) <0.001	1.03 (1.02, 1.05) <0.001	1.02 (1.00, 1.03) <0.001
**TyG-WC quartile**				
**Q1**	134 (16.90)	Ref	Ref	Ref
**Q2**	168 (21.21)	1.24 (1.02, 1.51) 0.032	1.24 (1.02, 1.50) 0.035	1.26 (1.03, 1.55) 0.033
**Q3**	174 (21.97)	1.33 (1.09, 1.61) 0.004	1.31 (1.08, 1.59) 0.007	1.37 (1.13, 1.67) 0.027
**Q4**	214 (26.99)	1.60 (1.33, 1.93) <0.001	1.60 (1.32, 1.93) <0.001	1.67 (1.38, 2.03) <0.001
**DM**				
**TyG-WC (per 20 units)**	250 (25.43)	1.05 (1.03, 1.07) <0.001	1.05 (1.03, 1.07) <0.001	1.03 (1.00, 1.05) <0.001
**TyG-WC quartile**				
**Q1**	38 (15.45)	Ref	Ref	Ref
**Q2**	59 (24.08)	1.57 (1.09, 2.25) 0.014	1.56 (1.09, 2.24) 0.016	1.98 (1.04, 3.75) 0.037
**Q3**	69 (28.05)	1.76 (1.24, 2.51) 0.002	1.71 (1.20, 2.44) 0.003	2.15 (1.15, 4.01) 0.016
**Q4**	84 (34.15)	2.36 (1.68, 3.32) <0.001	2.37 (1.68, 3.34) <0.001	2.44 (1.31, 4.55) 0.005

Model 1: Unadjusted.

Model 2: Adjusted for age, sex, education level, and marital status.

Model 3: Adjusted for age, sex, education level, marital status, SBP, DBP, BMI, social activity, residence, HbA1c, HDL-C, LDL-C, BUN, UA, drinking history, smoking history, dyslipidemia, kidney disease, and history of medication use.

Table S11 Association between TyG-WC and CVD after excluding individuals with missing covariates.

Categories	Event, n (%)	Model 1 *HR* (95% *CI*) *P* value	Model 2 *HR* (95% *CI*) *P* value	Model 3 *HR* (95% *CI*) *P* value
**TyG-WC (per 20 units)**	1528 (20.31)	1.04 (1.03, 1.05) <0.001	1.04 (1.03, 1.04) <0.001	1.01 (1.00, 1.02) <0.001
**TyG-WC quartile**				
**Q1**	298 (15.84)	Ref	Ref	Ref
**Q2**	334 (17.76)	1.14 (1.02, 1.28) 0.018	1.15 (1.03, 1.29) 0.012	1.13 (1.01, 1.26) 0.034
**Q3**	411 (21.85)	1.43 (1.23, 1.66) <0.001	1.41 (1.22, 1.64) <0.001	1.25 (1.07, 1.47) 0.006
**Q4**	539 (28.64)	1.94 (1.68, 2.23) <0.001	1.91 (1.66, 2.20) <0.001	1.46 (1.23, 1.73) <0.001

Model 1: Unadjusted.

Model 2: Adjusted for age, sex, education level, and marital status.

Model 3: Adjusted for age, sex, education level, marital status, SBP, DBP, BMI, social activity, residence, HbA1c, HDL-C, LDL-C, BUN, UA, drinking history, smoking history, diabetes, dyslipidemia, kidney disease, and history of medication use.

Table S12 Association between TyG-WC and the risk of CVD according to glucose metabolic states after excluding individuals with missing covariates.

Categories	Event, n (%)	Model 1 *HR* (95% *CI*) *P* value	Model 2 *HR* (95% *CI*) *P* value	Model 3 *HR* (95% *CI*) *P* value
**NGR**				

**TyG-WC (per 20 units)**	585 (18.80)	1.03 (1.02, 1.05)<br><0.001	1.03 (1.02, 1.05)<br><0.001	1.02 (1.00, 1.03)<br>0.004
**TyG-WC quartile**				
**Q1**	125 (12.07)	Ref	Ref	Ref
**Q2**	115 (14.78)	1.16 (0.91, 1.47)<br>0.227	1.11 (0.87, 1.41)<br>0.402	1.08 (0.84, 1.39)<br>0.540
**Q3**	143 (18.38)	1.31 (1.05, 1.65)<br>0.019	1.21 (0.96, 1.52)<br>0.112	1.09 (0.86, 1.38)<br>0.489
**Q4**	202 (25.96)			
**Pre-DM**				
**TyG-WC (per 20 units)**	727 (21.87)	1.04 (1.03, 1.05)<br><0.001	1.02 (1.01, 1.04)<br><0.001	1.02 (1.00, 1.03)<br><0.001
**TyG-WC quartile**				
**Q1**	140 (16.85)	Ref	Ref	Ref
**Q2**	178 (21.42)	1.30 (1.04, 1.63)<br>0.019	1.29 (1.03, 1.61)<br>0.025	1.29 (1.03, 1.61)<br>0.026
**Q3**	181 (21.78)	1.32 (1.06, 1.65)<br>0.014	1.27 (1.01, 1.58)<br>0.038	1.26 (1.01, 1.58)<br>0.044
**Q4**	228 (27.44)	1.70 (1.38, 2.10)<br><0.001	1.55 (1.25, 1.93)<br><0.001	1.57 (1.26, 1.97)<br><0.001
**DM**				
**TyG-WC (per 20 units)**	270 (24.79)	1.04 (1.03, 1.05)<br><0.001	1.03 (1.01, 1.05)<br><0.001	1.03 (1.01, 1.05)<br><0.001
**TyG-WC quartile**				
**Q1**	42 (15.44)	Ref	Ref	Ref
**Q2**	59 (21.69)	1.66 (1.25, 2.19)<br><0.001	1.44 (1.09, 1.92)<br>0.011	1.38 (1.04, 1.84)<br>0.027
**Q3**	77 (28.31)	2.21 (1.69, 2.90)<br><0.001	1.78 (1.35, 2.35)<br><0.001	1.70 (1.28, 2.25)<br><0.001
**Q4**	92 (33.70)	2.63 (2.02, 3.42)<br><0.001	1.81 (1.37, 2.39)<br><0.001	1.83 (1.35, 2.47)<br><0.001

Model 1: Unadjusted.

Model 2: Adjusted for age, sex, education level, and marital status.

Model 3: Adjusted for age, sex, education level, marital status, SBP, DBP, BMI, social activity, residence, HbA1c, HDL-C, LDL-C, BUN, UA, drinking history, smoking history, dyslipidemia, kidney disease, and history of medication use.
